# Acceptance of virtual dental implant planning software in an undergraduate curriculum: a pilot study

**DOI:** 10.1186/1472-6920-12-90

**Published:** 2012-09-29

**Authors:** Emeka Nkenke, Elefterios Vairaktaris, Anne Bauersachs, Stephan Eitner, Alexander Budach, Christian Knipfer, Florian Stelzle

**Affiliations:** 1 Department of Oral and Maxillofacial Surgery, Erlangen University Hospital, Erlangen, Germany; 2 Department of Oral and Maxillofacial Surgery, University of Athens Medical School, Attikon Hospital, Athens, Greece; 3 Department of Prosthodontics, Erlangen University Hospital, Erlangen, Germany

## Abstract

**Background:**

Advances in healthcare such as virtual dental implant planning have the capacity to result in greater accuracy, speed, and efficiencies leading to improvement in patient care. It has been suggested that the acceptance of new technology is influenced by a variety of factors including individual differences, social and situational influences, user beliefs, and user attitudes. Despite the large volume of work in this area, only limited research has been conducted in the field of dental education. Therefore, the present study aimed at assessing the acceptance of virtual dental implant planning software by undergraduate students.

**Methods:**

Forty-three third-year dental students of the University of Erlangen-Nuremberg, Germany, were included in the study. They filled in a questionnaire based on a combination of the technology acceptance model and the theory of planned behavior (C-TAM-TPB). Cronbach’s α, Pearson product moment correlation coefficients, and squared multiple correlations (R^2^) were calculated.

**Results:**

Cronbach’s α exceeded .7 for all constructs. Pearson correlations were significant for the pairs perceived usefulness/behavioral intention, perceived usefulness/attitude, and attitude/behavioral intention. Perceived ease of use explained .09% of the variance of perceived usefulness (R^2^ = .09). Perceived ease of use and perceived usefulness accounted for 31% of the variance of attitude (R^2^ = .31). Perceived usefulness, attitude, subjective norm, and perceived behavioral control explain 37% of the variance of behavioral intention (R^2^ = .37).

**Conclusions:**

Virtual dental implant planning software seems to be accepted by dental students especially because of its usefulness and the students’ attitude towards this technology. On the other hand, perceived ease of use does not play a major role. As a consequence, the implementation of virtual dental implant planning software in a dental undergraduate curriculum should be supported by highlighting the usefulness by the supervisors, who should also strengthen the attitude of the students towards this technology.

## Background

Implant dentistry is one of the most dynamically evolving fields in oral healthcare. Dental implant treatment has become widely performed and documented. As a consequence, implants are firmly established as a part of mainstream dentistry [1]. Following significant expansion of indications for implant treatment, the recent advances in implant technology and treatment modalities have resulted in a rapid increase in demand from the public for such treatment. Oral healthcare professionals will increasingly encounter patients restored with dental implants, provide dental care and maintenance for them, or treat new patients seeking implant treatment. A modern curriculum should adequately prepare dental students with knowledge and competencies in implant dentistry [2].

The potential of three-dimensional (3D) simulation software for dental implant planning in an undergraduate setting has been recognized previously [3]. These tools are based on data acquired by conventional computed tomography or cone beam CT. Different sections of the radiographic data are visualized and matched with a 3D view of the scanned object. It is possible to take a closer look at different anatomical structures, zooming, rotating, and slicing aspects of the 3D object. Dental implants can be virtually planned, placed, and restored according to a chosen treatment plan and positioned related to anatomical and occlusal information. The planning itself can be saved and left for evaluation with the supervisor [3].

Advances in healthcare like virtual dental implant planning have the capacity to result in greater accuracy, speed, and efficiencies leading to improvement in patient care [4]. Universities, hospitals, and private practices spend relevant amounts of resources in new technology. Actually, the adoption of innovations is a critical investment decision. Simply acquiring technology is not a sufficient prerequisite for its effective utilization. The diffusion of technological innovations is dependent upon social processes. The assessment of sustainability of technological innovations needs to consider not only the technology itself but the manner in which these innovations alter the context in which healthcare transactions occur [5]. Understanding the conditions in which technology will be welcomed with open arms is of high importance. Low usage of innovations is a major focus of technology acceptance research [6]. Over the past decades, technology acceptance has been researched from multiple theoretical perspectives [7]. It has been suggested that acceptance behavior is influenced by a variety of factors including individual differences, social and situational influences, user beliefs, user attitudes, and managerial interventions. Despite the large volume of work in this area, only limited research has been conducted in the field of dental education [8]. Therefore, the present study aimed at assessing the acceptance of virtual dental implant planning software by undergraduate students.

## Methods

The study was approved by the institutional Ethics Committee of the University of Erlangen-Nuremberg. Forty-three third-year dental students (30 female, 13 male) were included in the study. They were scheduled for a dento-maxillofacial radiology course. The course was delivered as 28 face-to-face modules of 45 minutes. It included an introduction to the virtual implant planning software of two modules of 45 minutes with hands-on training (NobelClinician, Nobel Biocare AG, Kloten, Switzerland). Subsequently, each student received a copy of the software for installation on his or her personal computer. Over a period of three months the students had to carry out the planning of implant positions for the replacement of missing teeth in three different computed tomography data sets. Each planning was checked by a supervisor and feedback was given.

In order to gather information on the acceptance of the virtual implant planning software, a questionnaire was put together that was adapted from the technology acceptance model (TAM) and the theory of planned behavior (TPB) [9]. The questionnaire was based on the combined TAM and TPB (C-TAM-TPB). The items were measured on a six-point Likert scale ranging from 1 (“I totally disagree”) to 6 (“I totally agree”).

One week after the end of the dento-maxillofacial radiology course the students were asked to fill in the questionnaire. Participation was voluntary. Each individual consented to participate in the study. Demographic data of the participants was obtained.

The questionnaire included the subscales 1) perceived usefulness (PU), 2) perceived ease of use (PEU), 3) perceived behavioral control (PBC), 4) subjective norm (SN), 5) attitude (A), and behavioral intention (intention to use, BI). The definitions of the six constructs are given in Table 1. In technology acceptance models, perceived ease of use, subjective norm, and perceived behavioral control are independent constructs. Perceived usefulness and attitude are mediating constructs. Both constructs are dependent on perceived ease of use. Behavioral intention is a dependent construct. It is dependent on perceived usefulness, attitude, subjective norm, and perceived behavioral control.

**Table 1:** Definitions of the different constructs used in the present study

**Construct**	**Definition**
Perceived usefulness (PU)	Extent to which a student believes the software will improve his or her ability to perform dental implant planning
Perceived ease of use (PEU)	Extent to which a student believes using the dental implant planning software will improve his or her performance
Perceived behavioral control (PBC)	Students’ beliefs about the presence or absence of requisite resources and opportunities that influence using the implant planning software
Subjective norm (SN)	Students’ perception of what other people, who are important to them, feel about adopting the dental implant planning software
Attitude (A)	Students’ positive and negative feelings using the dental implant planning software
Behavioral intention (intention to use, BI)	Students’ beliefs about expected utilization of the dental implant planning system

The questionnaire consisted of 28 items, which are given in Table 2.

**Table 2:** Questionnaire and related results

**Construct**	**Cronbach’s α**	**Measurement items**	**Median**	**IQR**
Perceived usefulness	.85	The implant planning software enables me to accomplish tasks more quickly.	3	1
		The implant planning software has improved my quality of work.	4	2
		The implant planning software makes it easier to do my job.	3	1
		The implant planning software has improved my productivity.	5	3
		The implant planning software gives me greater control over my job.	4	2
		The implant planning software enhances my effectiveness on the job.	4	1
Perceived ease of use	.77	My interaction with the implant planning software has been clear and understandable.	2	3
		Overall, the implant planning software is easy to use.	2	1
		Learning to operate the implant planning software was easy for me.	2	1
		I rarely become confused when I use the implant planning software.	3	1
		I rarely make errors when using the implant planning software.	2	1
		I am rarely frustrated when using the implant planning software.	4	2
Perceived behavioral control	.73	I am able to confidently use the implant planning software.	4	2
		I have the knowledge to use the implant planning software.	3	1
		I have the resources to use the implant planning software.	6	2.5
		I have the ability to use the implant planning software.	5	1
		I have control over using the implant planning software.	5	1
Subjective norm	.73	People who influence my behavior think I should use the implant planning software.	5	3
		People who are important to me think I should use the implant planning software.	5	1
		My immediate supervisor thinks I should use the implant planning system.	6	1
		My close friends think I should use the implant planning system.	4	2
		My peers think I should use the implant planning system.	4	1
		People whose opinions I value prefer that I use the implant planning software in my work.	4	1
Attitude	.74	Using the implant planning software is a good idea.	5	.5
		Using the implant planning software is unpleasant.	2	2.5
		Using the implant planning software is beneficial to patient care.	5	1.5
Behavioral intention	.71	I intend to continue using the implant planning software to perform my job.	5	3
		I intend to frequently use the implant planning software to perform my job.	5	3

### Statistical analysis

Median values and interquartile ranges (IQR) are given for the results of the questionnaire. For the demographic data mean values with standard deviations are given. The binomial distribution was used to calculate if there was a significant difference in gender distribution. The Kruskal-Wallis test was adopted to assess if there was a statistical difference in age between female and male patients.

Cronbach’s α analysis was carried out to assess reliability of the questionnaire. Cronbach’s α coefficients were calculated for all of the subscales (perceived ease of use, perceived usefulness, subjective norm, attitude perceived behavioral control, behavioral intention). Cronbach’s α-values of .7 or higher are in the acceptable range recommended by the literature [10]. These values indicate that items are measuring the same concept. Alpha values above .8 reflect a high reliability.

Pearson product moment correlation coefficients were calculated. This type of correlation analysis is based on the categorical description of the variables being compared. Squared multiple correlations (R^2^) were calculated for the endogenous dependent variables to indicate the amount of variance explained or accounted for by the set of independent predictor variables.

P-values less than or equal to .05 were considered significant. All calculations were done using SPSS Version 14.0 for Windows (SPSS, Chicago, USA).

## Results

The study population consisted of significantly more female than male students (p = .014). The age of females (24.37 ± 2.08 years, minimum 22 years, maximum 30 years) and males (24.92 ± 2.72 years, minimum 22 years, maximum 31 years) did not differ statistically significantly (p = .627).

All 43 questionnaires were filled in completely and could be used for data analysis. The results for the different items are given in Table 2. Cronbach’s α exceeded .7 for all constructs (Table 2).

Table 3 includes the data for the correlation between the relevant pairs of independent and dependent constructs. Pearson correlations were significant for the pairs perceived usefulness (independent construct)/behavioral intention (dependent construct), perceived usefulness (independent construct)/attitude (dependent construct), and attitude (independent construct)/behavioral intention (dependent construct).

**Table 3:** Pearson correlations for different combinations of the constructs

**Independent variable**	**Dependent variable**	**Pearson correlation (p-value)**
Perceived ease of use	Perceived usefulness	.255 (.099)
Perceived ease of use	Attitude	.276 (.073)
Perceived usefulness	Attitude	.450 (.002)
Perceived usefulness	Behavioral intention	.495 (.001)
Attitude	Behavioral intention	.402 (.008)
Subjective norm	Behavioral intention	.287 (.071)
Perceived behavioral control	Behavioral intention	.179 (.250)

Perceived ease of use explained .09% of the variance of perceived usefulness (R^2^ = .09). Perceived ease of use and perceived usefulness accounted for 31% of the variance of attitude (R^2^ = .31). Perceived usefulness, attitude, subjective norm, and perceived behavioral control explain 37% of the variance of behavioral intention (R^2^ = .37).

## Discussion

The acceptance of new technologies is a prominent problem in the healthcare arena. The attitude of target users towards innovations plays a pivotal role. End users will decide to use or misuse them, to incorporate them into their routine or work around them [11]. The use of new technologies is expected to steadily increase in healthcare including dentistry. With technological advances comes the challenge of how best to use them in dental education. Educational researchers are challenged to test the effectiveness and efficiencies of these new methods [12]. The ability to identify, predict, and manage acceptance of technology will facilitate implementation efforts. Therefore, the present study aimed at assessing the acceptance of virtual dental implant planning software by undergraduate students.

In the past, different models have been developed to predict and explain the end-user reactions to new technologies [13]. The technology acceptance model and the extended technology acceptance model have been tailored to the information systems context [6]. However, the technology acceptance models have not been developed specifically in or for the healthcare context. If used in their generic form, they may not capture or may contradict some of the unique contextual features of computerized healthcare delivery. Therefore, several additions and modifications have been made to adapt the technology acceptance models to the healthcare arena [11]. Today, they have widespread application in explaining healthcare providers’ reactions to new technologies. The increase in the use of the technology acceptance models appears justified. Many of the relationships specified by technology acceptance models have been repeatedly validated in healthcare settings [11].

An alternative model in the acceptance of new technology is the theory of planned behavior [14]. Its validity has been demonstrated in the healthcare sector [13]. It is theorized that perceived behavioral control is an additional important determinant of behavioral intention that is missing in the technology acceptance models. Consequently, the technology acceptance model and the theory of planned behavior have been combined in an integrated model, which was successfully used in the healthcare sector [9]. In order to be able to make use of all the different constructs the combined model (C-TAM-TPB) was adopted in the present study.

The reliability of the measurements of the different constructs in the present study compared well to other trials on technology acceptance in different fields of healthcare. Cronbach’s α values ranging from .55 to .93 have been described by different authors [9,15,16]. The range of the Cronbach’s α values was not that pronounced in the present study (.71–.85, Table 2). However, the Cronbach’s α values were always above .7, indicating an acceptable reliability [10].

In the present study C-TAM-TPB did not include demographic data. Significantly more female students were included and the age range was small. Therefore, it was decided not to base the analysis on these aspects. Previous studies on acceptance of technology have already shown the limited relevance of demographic data in the healthcare context [15].

In the present study perceived usefulness showed a significant correlation to attitude (p = .002) as well as behavioural intention (p = .001, Table 3). As with previous studies perceived usefulness appeared to be one of the most important factors affecting the students’ acceptance of the new technology [15]. On the other hand, perceived ease of use did not show a significant correlation with either perceived usefulness or attitude (Table 3). It seems that dental students are pragmatic in their technology acceptance decisions, appearing to focus on usefulness in technology assessment. They tend to accept a technology when it is considered to be useful to their practice independent of whether the use of the technology is convenient. This finding is consistent with the results of previous studies that showed that usefulness is more important than ease of use [17].

Such results have especially been found for physicians. It has been hypothesized that physicians have relatively high general competence and mental/cognitive capacity and may comprehend the use of a technology quickly. They seem to become familiar with operations of new technologies without going through the intense training that might be necessary among other user populations [15]. The same might be true for dental students. It seems that dental students will be able to successfully face the future challenges of implementation of new technologies in undergraduate curricula.

At the Dental School of the University of Erlangen-Nuremberg students are confronted with implant dentistry from the first day on. They gather profound theoretical knowledge in the field. Moreover, the students have the possibility of observing implant surgery and treating selected patient cases prosthodontically by themselves. However, so far there was a gap as far as the use of the acquired knowledge on implant dentistry for treatment planning was concerned. The use of virtual implant planning software in an undergraduate setting seems to be a relevant solution for this problem [18]. The students are put into a position where they can plan implant treatment and virtually place and restore these implants. They are enabled to transfer their theoretical knowledge to a more practical situation. It can be assumed that the addition of virtual implant planning to an undergraduate curriculum leads to a deeper understanding of implant dentistry. Providing copies of the software to the students enables them to use the virtual dental implant planning tool at their convenience as long as they want to. Getting feedback from the supervisors on the quality of the planned patient cases leads to an additional increase of the learning effect and supports further reflection on the topic [19]. As a consequence, the good acceptance of the virtual dental implant planning software by the students in the present study does not seem be surprising.

The major limitation of the study is the sample size. It does not allow use of structural equation modelling to analyze causal relations between model parameters. For the estimation of the parameters it is necessary to adopt a maximum likelihood estimation, which requires a sample size of at least 100 [15].

## Conclusions

Virtual dental implant planning software seems to be accepted by dental students because of its usefulness and the students’ attitude towards this technology. As a consequence, the implementation of virtual dental implant planning software in a dental curriculum should be supported by highlighting the usefulness by the supervisors who should also strengthen the attitude of the students towards this technology.

## Competing interests

The authors declare that they have no competing interests.

## Authors’ contributions

EN led on conception, design, statistical analysis, and interpretation of the data. EV contributed to drafting and revising of the article and critically appraising the content. SE contributed to drafting and revising of the article and critically appraising the content. AB was responsible for design and execution of the study. CK contributed to execution of the study and drafting of the manuscript. FS led on conception, design, statistical analysis, and interpretation of the data. All authors have approved the final version of the article submitted.

## Pre-publication history

The pre-publication history for this paper can be accessed here:

http://www.biomedcentral.com/1472-6920/12/90/prepub
